# Structural Plasticity Denoises Responses and Improves Learning Speed

**DOI:** 10.3389/fncom.2016.00093

**Published:** 2016-09-08

**Authors:** Robin Spiess, Richard George, Matthew Cook, Peter U. Diehl

**Affiliations:** ^1^Department of Computer Science, Swiss Federal Institute of Technology (ETH Zurich)Zurich, Switzerland; ^2^Institute of Neuroinformatics, ETH Zurich and University ZurichZurich, Switzerland

**Keywords:** structural plasticity, STDP, learning, spiking neural network, homoeostasis

## Abstract

Despite an abundance of computational models for learning of synaptic weights, there has been relatively little research on structural plasticity, i.e., the creation and elimination of synapses. Especially, it is not clear how structural plasticity works in concert with spike-timing-dependent plasticity (STDP) and what advantages their combination offers. Here we present a fairly large-scale functional model that uses leaky integrate-and-fire neurons, STDP, homeostasis, recurrent connections, and structural plasticity to learn the input encoding, the relation between inputs, and to infer missing inputs. Using this model, we compare the error and the amount of noise in the network's responses with and without structural plasticity and the influence of structural plasticity on the learning speed of the network. Using structural plasticity during learning shows good results for learning the representation of input values, i.e., structural plasticity strongly reduces the noise of the response by preventing spikes with a high error. For inferring missing inputs we see similar results, with responses having less noise if the network was trained using structural plasticity. Additionally, using structural plasticity with pruning significantly decreased the time to learn weights suitable for inference. Presumably, this is due to the clearer signal containing less spikes that misrepresent the desired value. Therefore, this work shows that structural plasticity is not only able to improve upon the performance using STDP without structural plasticity but also speeds up learning. Additionally, it addresses the practical problem of limited resources for connectivity that is not only apparent in the mammalian neocortex but also in computer hardware or neuromorphic (brain-inspired) hardware by efficiently pruning synapses without losing performance.

## 1. Introduction

To date, numerous models have been proposed to capture the learning process in the mammalian brain. Many of them focus on synaptic plasticity which describes the change of the synaptic state. Even though the creation and pruning of synapses (structural plasticity) is not only a key feature during development but also in the adult brain (Majewska et al., 2006; Holtmaat and Svoboda, 2009), modeling of structural plasticity has received less attention. Specifically, there is little literature on the interaction between the two plasticity processes, which is of major importance when trying to understand learning.

### 1.1. Structural plasticity

First findings that structural plasticity plays a role in the human development date back to 1979. Huttenlocher found that synaptic density increases during infancy, reaching a maximum at age 1–2 years which was about 50% above the adult mean. The decline in synaptic density observed between ages 2–16 years was also accompanied by a slight decrease in neuronal density (Huttenlocher, 1979). Also in the mature brain connections are pruned and new ones are created. The percentage of stable dendritic spines, which are part of most excitatory synapses, in adult mice are thought to be between 75 and 95% over 1 month (Holtmaat and Svoboda, 2009).

Experience-dependent structural plasticity often happens in tandem with synaptic plasticity (Butz et al., 2009). In other words, long-term potentiation (LTP) and long-term depression (LTD) might be closely related to structural rewiring. While synaptic efficacies change within seconds, structural rewiring might be more important on larger timescales (Chklovskii et al., 2004). It has been shown that presynaptic activity and glutamate can trigger spine growth and increases connectivity (Maletic-Savatic et al., 1999; Richards et al., 2005; Le Bé and Markram, 2006). Thus, new synapses are preferentially formed next to already existing synapses which were enhanced by long-term potentiation (LTP) (Engert and Bonhoeffer, 1999; Toni et al., 1999). Synapses weakened by LTD are more likely to be deleted (Ngerl et al., 2004; Le Bé and Markram, 2006; Becker et al., 2008).

### 1.2. Previous work on modeling structural plasticity

Even though the existence of structural plasticity has been known for quite some time, work on computational modeling of structural plasticity is still scarce.

Mel investigated the importance of spatial ordering and grouping of synapses on the dendrite (Mel, 1992). Learning included the rearrangement of the synapses. This enabled a neuron to learn non-linear functions with a single dendritic tree.

Butz and van Oyen have developed a rule for synapse creation based on axonal and dendritic elements (Butz and van Ooyen, 2013). Two neurons form a connection with a probability based on their distance from each other, and on the number of free and matching axonal boutons and dendritic spines. The axonal and dendritic elements were also created and deleted based upon the electrical activity of the neuron to reach a desired level of activity (the homeostatic set-point). Applied on a simulation of the visual cortex after focal retinal lesion their model produces similar structural reorganizations as observed in experiments. In a later publication they also show that the same rule can increase the performance and efficiency of small world networks (Butz et al., 2014).

Bourjaily and Miller modeled structural plasticity by replacing synapses which have too little causal correlation between pre- and post-synaptic spikes (Bourjaily and Miller, 2011). The replacement was done by choosing either a new pre- or post-synaptic neuron, while keeping the other one the same. They found that structural plasticity increased the clustering of correlated neurons which led to an increased network performance.

Poirazi and Mel present findings which show that the memory capacity provided by structural plasticity is magnitudes larger than that of synaptic plasticity (Poirazi and Mel, 2001). In other words, the synaptic weights are not the only or even the most important form of parameters which are used to store learned information. Also interesting is their finding of the benefit of large quantities of silent synapses. These silent synapses are potential candidates to replace eliminated synapses.

Hussain et al. implemented a model which clusters correlated synapses on the same dendritic branch with a hardware-friendly learning rule (Hussain et al., 2015). The proposed model attains comparable performance to Support Vector Machines and Extreme Learning Machines on binary classification benchmarks while using less computational resources.

Knoblauch et al. developed a model with “potential synapses” and probabilistic state changes (Knoblauch et al., 2014). They found that structural plasticity outperforms synaptic plasticity in terms of storage capacity for sparsely connected networks. Their theory of structural plasticity can also explain various memory related phenomena.

A global pruning rate of connections has been shown by Navlakha et al. to create more efficient and robust networks when starting with a highly connected network (Navlakha et al., 2015). The best results were obtained with a decreasing pruning rate, starting with many deletions followed by less and less pruning activity.

Other models also consider the creation of new neurons. For example the Spike-Timing-Dependent Construction algorithm by Lightheart et al. (2013) which models the iterative growth of a network. It produces similar results as STDP but also accounts for synapse and neuron creation.

### 1.3. Summary

In this study we explore what influence different structural plasticity mechanisms have when used in addition to spike-timing-dependent plasticity (STDP). Does the performance of the spiking neural network improve with the additional plasticity?

All of the structural plasticity mechanisms are based on weight changes induced by STDP, i.e., a lower synaptic weight will lead to an increased chance that the synapse is pruned. Additionally, we tested different strategies for synapse creation, either keeping the number of existing synapses constant or reducing them over time.

The structural plasticity mechanisms were tested on two different networks. The first network consists of one input population and one highly recurrently connected population that in turn consists of excitatory and inhibitory leaky integrate-and-fire neurons. We use a Gaussian-shaped input (with circular boundaries) and a population code (specifically the circular mean of the neuron activities). Using this network, we investigated the effect of structural plasticity on the neuron responses. The second network consists of four of the populations used in the first model. While three of those populations receive direct input from input populations, the fourth population only receives input from the other three recurrent populations (Diehl and Cook, 2016). Since this “three-way network” can (after learning) infer missing input values, it is useful for assessing the effect of structural plasticity on inference performance in terms of learning speed and precision of the inferred value.

For all tested structural plasticity mechanisms the quality of the signal increases, i.e., the amount of noise in the response is reduced compared to relying solely on STDP for learning. Similarly, the three-way network's inference performance increases *faster* when using structural plasticity (the performance after convergence is equal). Considering that additional connections also require additional resources such as physical space and energy, limiting the total number of connections is crucial for large-scale practical implementations. Therefore, the result that a reduction of the number of connections does not lead to a performance loss for the tested networks further corroborates the usefulness of structural plasticity.

This work shows that structural plasticity offers not only the possibility to improve the quality of the results but also to save resources. This applies to mammalian brains as well as simulating neural networks on traditional hardware and on brain-inspired neuromorphic hardware (Indiveri et al., 2006; Khan et al., 2008; Merolla et al., 2014). In biology the reduction of axons allows for less energy requirements and for thicker myelination in the course of development (Paus et al., 1999), in computer simulations the reduction of connections leads to less computation and it allows to adapt the number of connections in a neural network to the (often physically limited) connectivity in neuromorphic hardware.

## 2. Materials and methods

### 2.1. Model

The implementation of the model was done in python using the Brian library (Goodman and Brette, 2009).

#### 2.1.1. Leaky integrate-and-fire neuron

The model used for the neurons is the *leaky integrate-and-fire* model (Dayan and Abbott, 2001). Two different types of neurons are modeled: excitatory and inhibitory neurons. A leaky integrate-and-fire neuron fires a signal as soon as its membrane potential reaches a certain threshold *V*_thresh_. The signal travels to all connected neurons and influences them. Additionally the membrane potential of the firing neuron is reset to *V*_reset_. All parameter values are provided in Table 1.

(1) $$\begin{array}{ll} \frac{dV}{dt} & =\frac{(V_{\text{rest}}-V)+(I_{e}+I_{i})\frac{1}{\text{nS}}}{V_{\text{timeconstant}}} \end{array}$$

**Table 1:** **Parameters used to simulate the leaky integrate-and-fire neurons and those for the STDP rule**.

**Neuron parameter**	**Excitatory neuron**	**Inhibitory neuron**
*V*_rest_	−65 mV	−60 mV
*V*_reset_	−65 mV	−45 mV
*V*_thresh_	−52 mV	−40 mV
*V*_timeconstant_	20 ms	10 ms
**STDP parameter**	**Pre-synaptic**	**Post-synaptic**
ν	0.0005	0.0025
η	0.2	0.2
*w*_max_		0.5

The membrane potential is increased by the excitatory current *I*_*e*_ and decreased by the inhibitory current *I*_*i*_. But besides the excitatory and inhibitory current there is also a leak term. It slowly reverts the membrane potential back to the resting potential *V*_rest_. This leak term introduces a time dependency, since the incoming signals need to be close in time to accumulate and have the biggest influence on the potential.

(2) $$I_{e}\;=-V\;\cdot \;g_{e}\text{ nS}$$

(3) $$I_{i}\;=(-85\text{mV}-V)\;\cdot \;g_{i}\text{ nS}$$

(4) $$\frac{dg_{e}}{dt}\;=\frac{-g_{e}}{5\text{ ms}}$$

(5) $$\frac{dg_{i}}{dt}\;=\frac{-g_{i}}{10\text{ ms}}$$

The excitatory and inhibitory currents depend on the conductances *g*_*e*_ and *g*_*i*_ respectively. Depending on whether the neuron receives a signal from an excitatory or an inhibitory neuron the respective conductance increases temporarily. The simulation time step is 0.5 ms.

#### 2.1.2. STDP rule

The spike-timing-dependent plasticity (STDP) rule used for the simulations is largely based on the nearest spike model by Pfister and Gerstner (2006). This rule uses traces to keep track of the activity of the pre- and postsynaptic neuron. The trace *r* is set to 1 whenever the presynaptic neuron sends a spike. Another trace *o* is set to 1 when the postsynaptic neuron fires. Both *r* and *o* slowly decrease to zero over time. These traces are used to determine how much the weight *w* of the synapse should change.

Additionally a weight dependent term is multiplied to each equation. This prevents weights from going to the extreme values too fast. Larger weights decrease faster and increase slower while small weights do the opposite. With this term it is also possible to enforce a maximum strength *w*_max_. The specific parameter values are described in Table 1.

Equation (6) is applied to the synapse whenever the presynaptic neuron fires. The synapse's strength *w* is decreased based on the current weight, the freely adjustable parameter ν_pre_ and the parameter *o*. This means that the synapse is more weakened if the postsynaptic neuron has just fired and *o* is large.

(6) $$w\leftarrow w-o\;\cdot \;\nu_{\text{pre}}\;\cdot \;w^{\eta_{\text{pre}}}$$

When the postsynaptic neuron fires, *w* is increased according to Equation (7). It grows more if the presynaptic neuron has just fired as well i.e., *r* is large. It also depends on *o* which means that the weight is only increased if the postsynaptic neuron has spiked before. Note that *o* is set to 1 *after* this change is applied. The weight dependent term prevents connections from growing infinitely strong.

(7) $$w\leftarrow w+r\;\cdot \;\nu_{\text{post}}\;\cdot \;o\;\cdot \;{\left(w_{\text{max}}-w\right)}^{\eta_{\text{post}}}$$

The traces decay exponentially.

(8) $$\frac{do}{dt}\;=\frac{-o}{40\text{ ms}}\text{ For excitatory to excitatory synapses}$$

(9) $$\frac{do}{dt}\;=\frac{-o}{20\text{ ms}}\text{ For inhibitory to excitatory synapses}$$

(10) $$\frac{dr}{dt}\;=\frac{-r}{20\text{ ms}}\text{ For both types of synapses}$$

#### 2.1.3. Single population network

**Figure 1:**
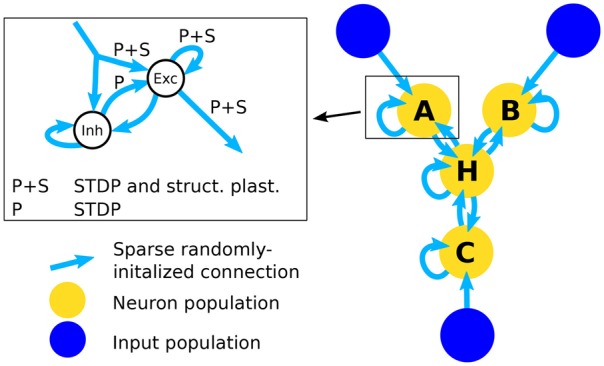
**Architecture of the network**. The right part of the figure shows the full network that is used in the simulations for testing inference performance. The simplified network that is used to assess the effect of structural plasticity on the amount of noise is composed of an input population and one neuron population that is connected to it. The inset shows the structure of a single neuron population. An input population consists of 1600 excitatory neurons that output Poisson-distributed spike-trains with firing rates determined by the stimulus value. A neuron population consists of 2000 neurons, 1600 of which are excitatory (Exc) and 400 are inhibitory (Inh). All possible types of recurrent connections within a population are present, i.e., Exc → Exc, Exc → Inh, Inh → Exc, Inh → Inh. Connections from Inh → Exc (denoted with “P”) use STDP and connections between excitatory neurons use STDP and structural plasticity (denoted with “P+S”). Note that long-range connections between populations are always originating from excitatory neurons and posses the same structure. Therefore the connections from input to neuron populations and connections between different neuron populations are not differentiated between in the inset.

In order to test the structural plasticity algorithms, a recurrent network model was used, as described in Diehl and Cook (2016). It consists of one input population and only a single computation population. The input population consists of 1600 excitatory neurons which are simulated as spike trains according to a Poisson distribution. Section 2.1.5 explains the shape of the input in more detail. The computation population has 1600 excitatory neurons and 400 inhibitory neurons. These neurons are leaky integrate-and-fire neurons. The connections within and between the groups of neurons can be seen in the inset of Figure 1. To initialize the connections 10% of the possible synapses in each connection are chosen. The synapses are chosen at random with the only constraint that each target neuron has the same amount of input connection i.e., each column in the connection matrix has the same number of non zero values. This constraint increases the stability of the network slightly. The weight value of each synapse is random between zero and a maximum value depending on the type of connection (See Table 2). The goal of the computation population is to learn the pattern of the input population. The performance is measured by how fast and accurate the pattern is learned.

**Table 2:** **Maximum synapse weight values for initialization**.

**Connection type**	**Value**
Input to excitatory (single population)	1.0
Input to excitatory (three-way network)	0.5
Input to inhibitory	0.2
Excitatory to excitatory	0.2
Excitatory to inhibitory	0.2
Inhibitory to excitatory	1.0
Inhibitory to inhibitory	0.4

#### 2.1.4. Three-way network

Using multiple of the neuron populations, it is possible to construct a three-way network (Diehl and Cook, 2016). It contains three input populations and four computation populations that are highly recurrently connected. The network structure is shown in Figure 1. The connections are initialized in the same way as for the Single Population network. Such a network can learn arbitrary relations with three variables like *A*+*B*−*C* = 0. If a trained network receives only two of the three inputs, it can infer the missing one, e.g., if the network receives *A* and *B* it can infer the value of *C*. Here we choose the accuracy of the inference as the performance metric for this network.

The populations A, B, C, and H each use the same setup as the single population network and the input populations X, Y, and Z are equivalent to the input population in the single population network. The main difference lies in the bidirectional connectivity between populations. Note that the connectivity is bidirectional on a population level but not on a neuron level since often connections between neurons from different populations form connections only in one direction. The bidirectional connectivity enables the four populations of the network to reach a consistent state. Note that the long-range connections arriving at a neuron population are represented by the same input connection in the inset of Figure 1 since they are identical in structure and the neuron population cannot differentiate between connections originating from input populations and neuron populations. This state is random in the beginning but converges toward a correct solution of the input relation after learning. How well this convergence works exactly corresponds to our inference accuracy. For further information see Diehl and Cook (2016).

#### 2.1.5. Encoding and decoding

During each simulation multiple input examples are shown to the network with a duration of 250 ms per example. We use Gaussian-shaped inputs with wrap-around in combination with a population code to encode and decode the value of a population (Georgopoulos et al., 1986; Pouget et al., 2000). The standard deviation of the input Gaussian is $\sigma =\frac{1}{12}$ and the mean is the input value.

The value represented by a population can be calculated by the circular mean *ā* of the activities of each neuron of the population:

(11) $$\bar{a}=\text{arg}\left(\sum_{j=1}^{1600}a_{j}\exp(i\;\cdot \;\frac{j}{1600}2\pi )\right)$$

where *a*_1_, …, *a*_1600_ are the activities of the neurons 1–1600.

### 2.2. Noise estimation

An important performance criterion we use is the amount of noise in a population's spike response. We estimate the noise *o*_noise_ by fitting a Gaussian with offset to the response of a population (Equation 12).

(12) $$G(x,a,\mu ,\sigma ,o_{\text{noise}})\;=\;a\exp\left(\frac{-{\left(x-\mu \right)}^{2}}{2\sigma^{2}}\right)+\max(0,o_{\text{noise}})$$

The maximum function prevents the offset *o*_noise_ from being negative. The initial guesses for the free parameters are:

(13) $$a\;=\;\frac{1}{\sigma_{\text{input}}\sqrt{2\pi }}$$

(14) $$\mu \;=\text{ arg}\left(\sum_{j=1}^{1600}s_{j}\exp(i\;\cdot \;\frac{j}{1600}2\pi )\right),$$

where *s*_*j*_ is the spike activity of neuron j

(15) $$\sigma \;=\sigma_{\text{input}}$$

(16) $$o_{\text{noise}}\;=0$$

The fitting itself is done by SciPy's curve_fit function which employs non-linear least squares to fit (Equation 12) to the spike response. The resulting value for *o*_noise_ is the white-noise amount in the response.

### 2.3. Structural plasticity algorithms

Through the course of this investigation multiple algorithms with increasing complexity were devised to model structural plasticity. While all of the presented algorithms were performed with multiple parameter setting to that the observed effects are not a coincidence, we are presenting the results for each algorithm with one set of parameters for brevity. All of them are based on the current connection matrices. The rows of such a matrix represent the source neurons and the columns are the target neurons. The value of the matrix entry indicates how strong the synapse is. During training these algorithms are applied after every 50 input examples.

#### 2.3.1. Basic idea and implementation

The simplest algorithm consists of periodically checking the synapses and deleting those whose strength is below a certain threshold. New synapses are randomly inserted into the connection matrix. Deleting the weakest synapses which have the least influence on the network makes intuitively sense. It is also backed by evidence in biology that unused and weakened synapses are prone to being removed (Ngerl et al., 2004; Le Bé and Markram, 2006; Becker et al., 2008; Butz et al., 2009). The notion of deleting the weak synapses remains the same for all other algorithms as well. While there is no theoretical framework for the spiking neuron model used in this work there have been findings with theoretical derivations for this reasoning for associative memory networks with Hebbian learning (Chechik et al., 1998).

#### 2.3.2. Bookkeeping

**Figure 2:**
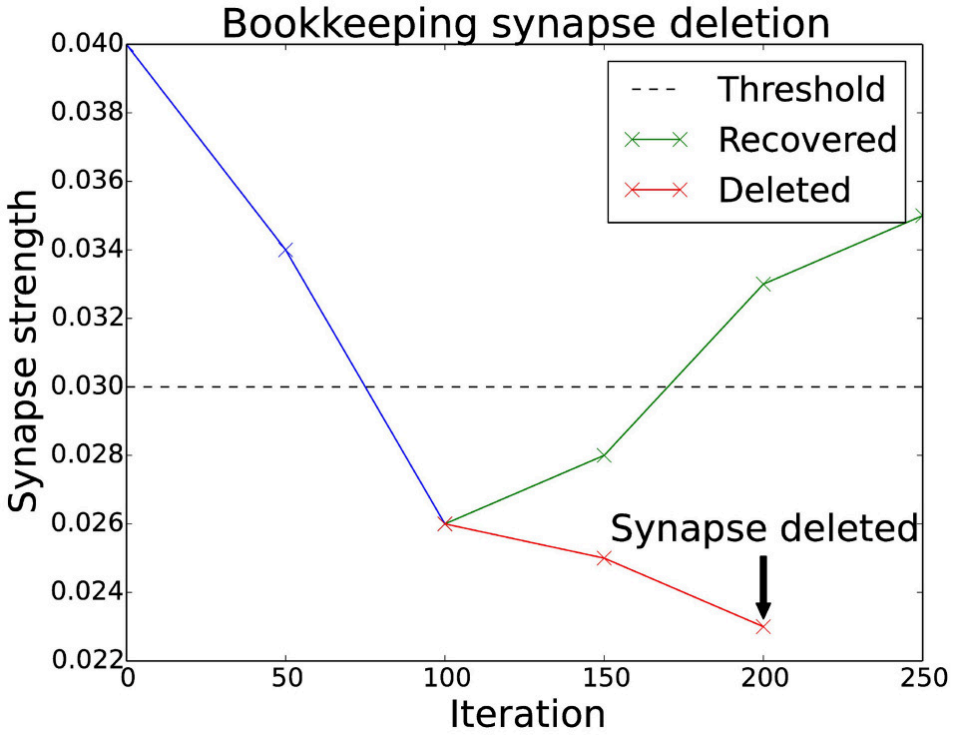
**Bookkeeping algorithm time schedule**. This plot shows two different scenarios for a weakened synapse. If its strength is under the threshold for an extended period, the synapse is deleted and replaced with a new one at a different location. The evaluation occurs as often as the bookkeeping algorithm is applied, which is after every 50 iterations. The number of times the synapse has been weaker than the threshold is stored in the bookkeeping matrix. The counter is slowly reset to zero if the synapse manages to recover its strength.

A generalization of the basic algorithm is to monitor the synapses over a period of time. If the synapse's strength is below a threshold, an entry in a sparse matrix (the “bookkeeping” matrix) is increased. As soon as the value in the bookkeeping matrix is larger than a certain threshold the synapse is finally deleted. Figure 2 shows how this plays out for a particular synapse. If the synapse recovers after being below the threshold, the entry in the bookkeeping matrix decreases until it is back at zero. This mechanism gives a much finer control over the deletion of synapses.

This mechanism also allows new synapses to have an additional margin of time before they are targeted for deletion. There have been biological findings which hint to such a period of grace (Le Bé and Markram, 2006). In the implementation this is simply done by using a negative value in the bookkeeping matrix.

**Figure 3:**
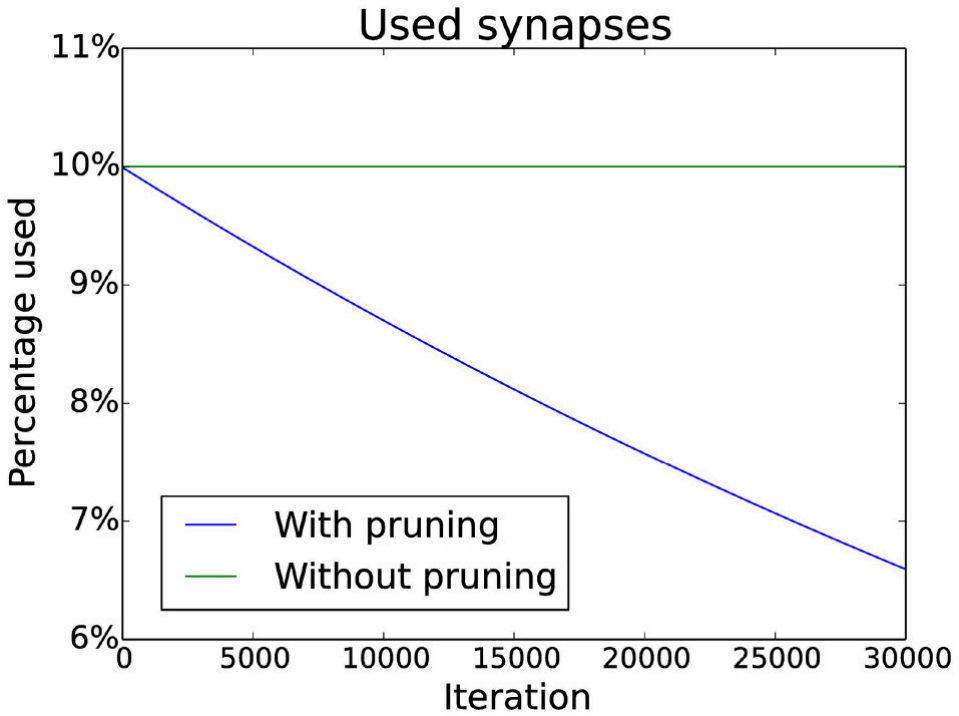
**Pruning schedule**. Initially 10% of the possible connections are used (without pruning this number does not change). With pruning the number of connections is decreased over time by multiplying the target number of connections by a constant factor smaller than one (effectively implementing an exponential decay).

New synapses are created in the same column of the connection matrix where they were deleted. This prevents starvation of neurons as it ensures that there are always input synapses for a neuron. Additionally, pruning of synapses can be simulated by slowly decreasing a target value for the number of synapses in each column. If there are too many synapses in a column, the weakest is deleted. The decrease follows an exponential decay which matches experimental data (Navlakha et al., 2015). Specific numbers can be seen in Figure 3.

#### 2.3.3. Including spatial information

Building on the bookkeeping algorithm additional spatial information is introduced. Instead of creating synapses uniformly at randomly in the same column, a probability distribution which depends on the existing synapses is used. It has been shown that presynaptic activity and glutamate can trigger spine growth and increases connectivity (Maletic-Savatic et al., 1999; Richards et al., 2005; Le Bé and Markram, 2006). Therefore the probability that new synapses are created is higher in the vicinity of already existing ones (Govindarajan et al., 2006; Butz et al., 2009).

**Figure 4:**
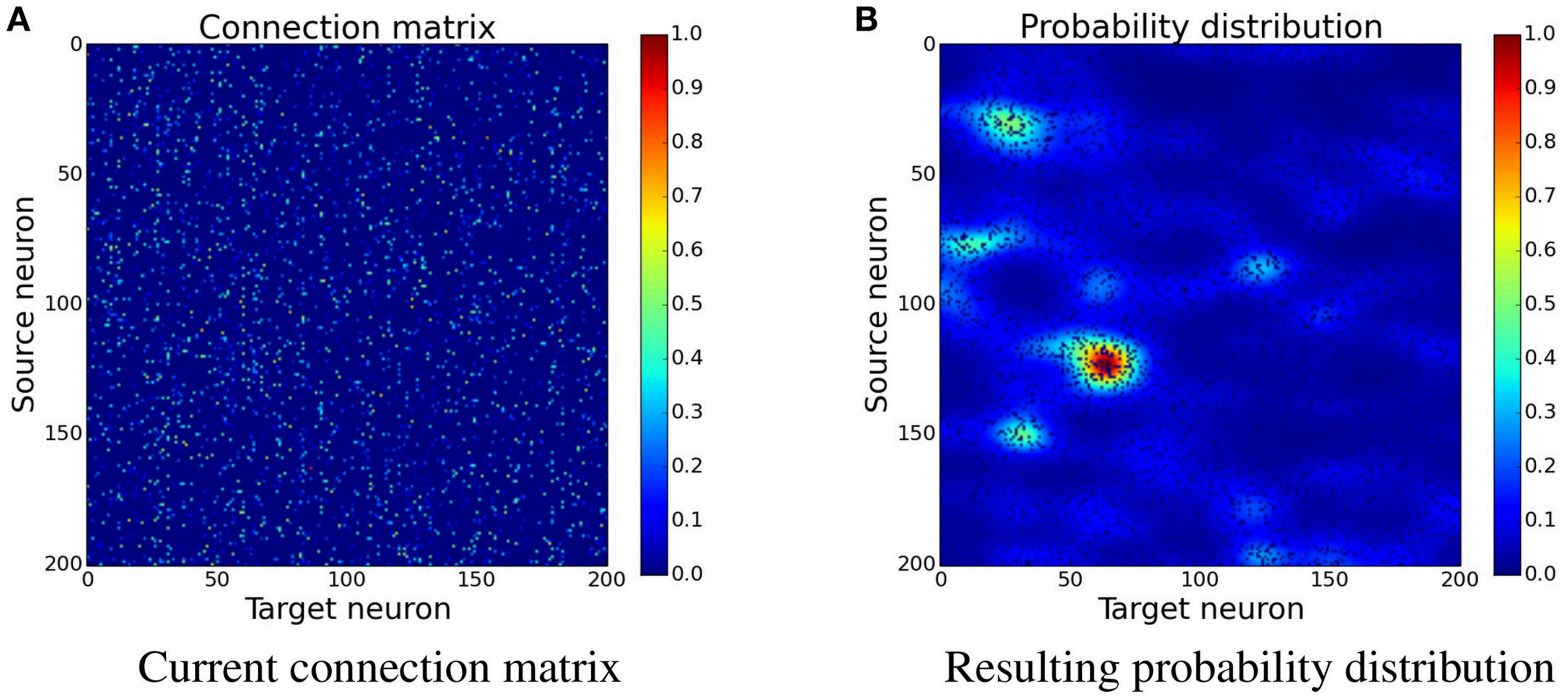
**Determining the position of new synapses**. The connection matrix is transformed into a probability distribution, which is not yet normalized, by using convolution with a two-dimensional Gaussian filter, see Equation (17). When choosing the place for a new synapse, locations with a higher value are more likely to be picked than the ones with a lower value. **(A)** Current connection matrix. **(B)** Resulting probability distribution.

The idea is to increase the probability for new synapses next to already existing synapses. In the implementation this is done by creating a custom probability distribution for the formation of new synapses. The probability is acquired by spreading the values of existing synapses to nearby free locations. An easy way to do this is convolving the connection matrix with a filter. Figure 4 shows how convolution with a Gaussian filter is used to transform the connection matrix into a probability distribution. Because convolution only creates the sum of the contributions, the resulting values are exponentiated in order to gain a multiplicative effect of nearby synapses. This leads to increased clustering of new synapses which has a positive effect as can be seen later in Section 3.3 and **Figure 10**.

(17) P=exp(W ∗ G)

where *W* is the current connection matrix containing the synapse strengths and *G* is a two-dimensional Gaussian filter with σ_horizontal_ = 10 and σ_vertical_ = 5. The larger horizontal standard deviation means that the Gaussian has a far reaching influence for the same source neuron but only a small influence on neighboring source neurons. The convolution is done with wrapped borders since the input is wrapped as well.

The final values *P* define the new probability distribution per column. The algorithm does the same steps as the bookkeeping algorithm, but instead of inserting new synapses at random within a column, it uses the custom probability distribution. This algorithm also decreases the total number of synapses over time with pruning which was introduced in the bookkeeping algorithm.

## 3. Results

### 3.1. Denoising of responses

**Figure 5:**
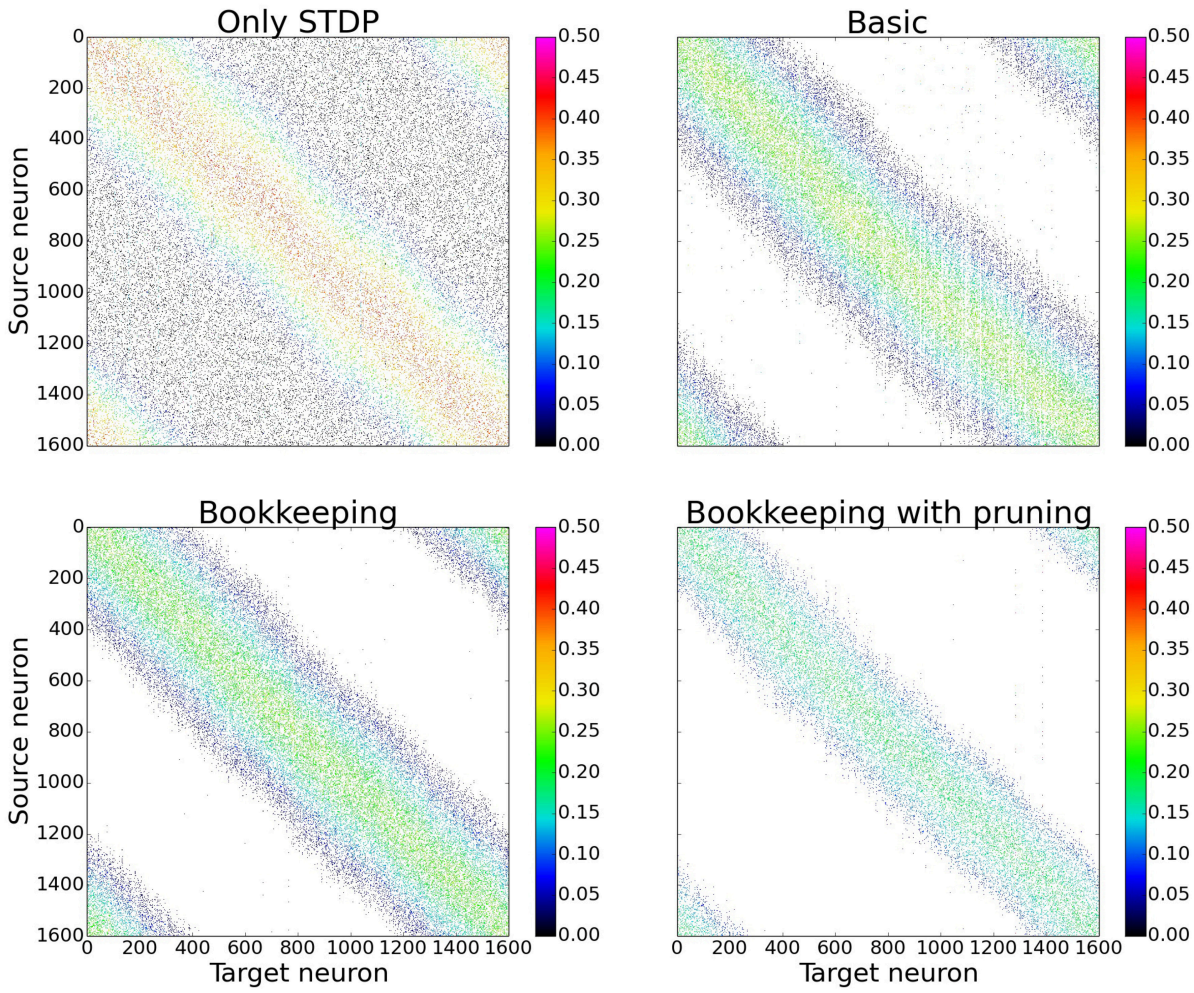
**Changes in topology for different structural plasticity mechanisms**. These are the connection matrices from the input population to the computation population for four differently trained networks. Since STDP has no mechanism to delete weak synapses the connection matrix of the network trained with only STDP has non zero entries spread evenly. Bookkeeping with pruning reduced the total number of synapses over time which led to a sparser matrix. The bottom left and upper right corner of each matrix have non-zero entries due to the wrap around and therefore periodic nature of the input.

In order to gain a first impression of the influence structural plasticity has during training, we use a single population network. When comparing the connection matrices that are only trained with STDP to the connection matrices of networks which additionally use structural plasticity, the main advantage of structural plasticity becomes apparent. Weak synapses which contribute mostly to the noise are removed. The columns of the connection matrices shown in Figure 5 are sorted according to their preferred input stimulus. Since the columns represent the target neuron of the synapses, each entry in a column is a synapse from a different source neuron. For the following performance measurements all matrices were sorted in that way (see Section 3.3 and **Figure 10** for results without this prior sorting). While the network that was trained only with STDP has non-zero entries that are distributed evenly, the networks using structural plasticity have all synapses concentrated on the preferred input.

**Figure 6:**
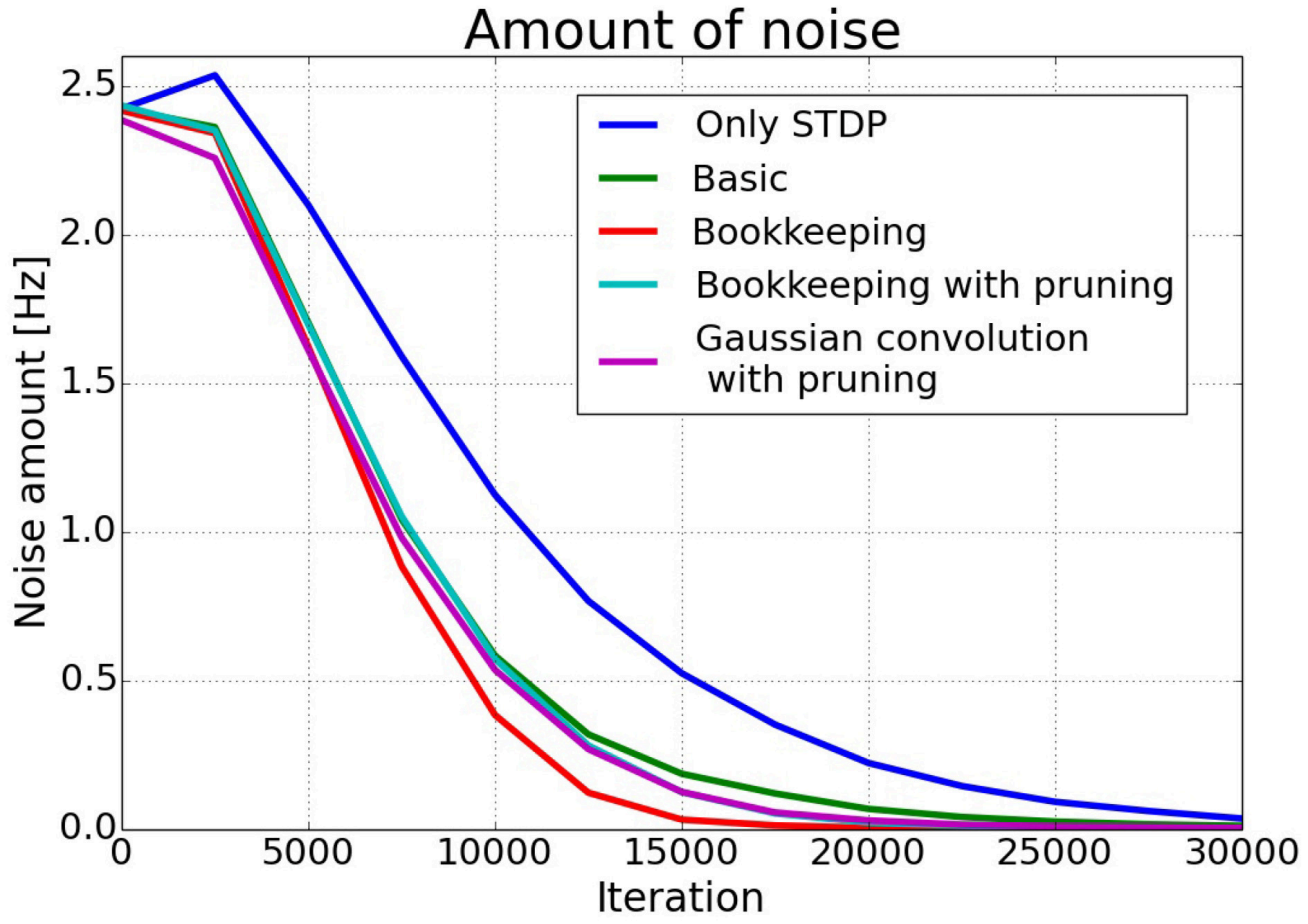
**Average noise amount**. The estimated noise amount *o*_noise_ as defined in Section 2.2. drops faster in networks trained with a structural plasticity algorithm. The different structural plasticity algorithms perform roughly equally well.

The effects of structural plasticity are also noticeable when computing the noise of the responses as described in Section 2.2. The graph in Figure 6 shows that structural plasticity decreases the noise amount faster. All structural plasticity algorithms perform roughly equally well. This shows that the details of the implementation are not that important in this case. The shown results are averaged over three different initial connection matrices. Each connection matrix was randomly initialized with the only constraint of having a certain number of non-zero entries per column, as described in more detail in Section 2.1.3.

**Figure 7:**
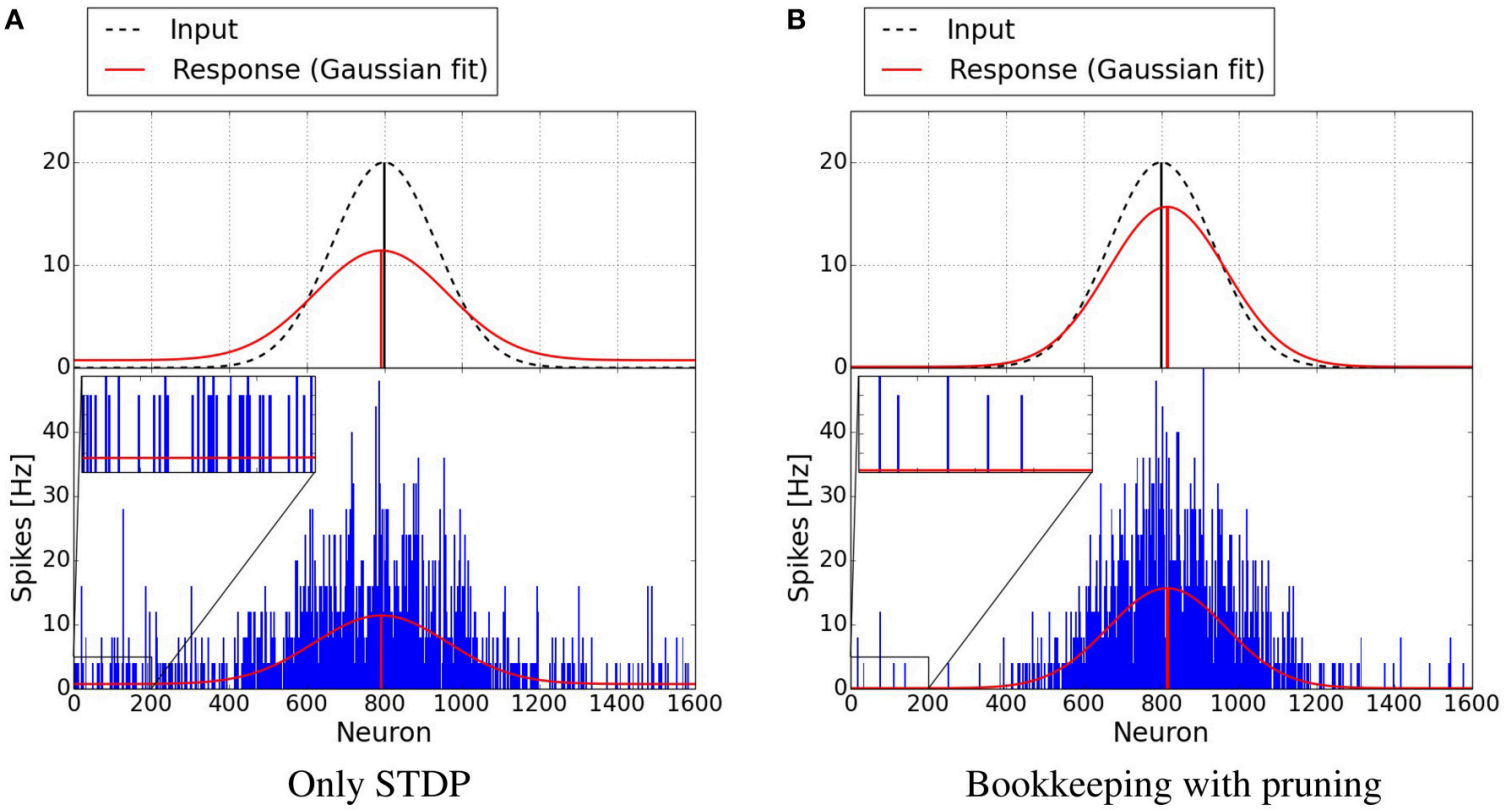
**Response of neuron populations to a single input examples**. The blue bars depict the frequency of spikes for each neuron. A Gaussian with an offset has been fitted to the spiking activity (red curve). The dashed Gaussian curves represent the input. Both networks were trained with 15,000 examples. The structural plasticity algorithm achieves a less noisy signal (*o*_noise_ = 0.0941, μ = 0.5092, σ = 0.0947) than the network trained with only STDP (*o*_noise_ = 0.7356, μ = 0.4949, σ = 0.1071). The input Gaussian has *o*_noise_ = 0, μ = 0.5 and σ = 0.0833. **(A)** Only STDP. **(B)** Bookkeeping with pruning.

Figure 7 shows plots of exemplary spike-responses with and without structural plasticity. The plots contain the spike response of two networks to an input example. Two Gaussian curves with an offset were fitted to these responses. The activity of the input population is also shown as a Gaussian. As Figure 6 suggests, the responses are less noisy when structural plasticity is used.

### 3.2. Improving learning speed

As a next step we investigate whether structural plasticity can improve the inference capabilities of the three-way network. The initial random connections are sorted once before training. The network is then trained with random inputs for populations *A* and *B* while *C* receives the input *A* + *B* modulo 1. During this training the network learns the relation *A* + *B* − *C* = 0. Here we test the performance of the network by measuring how well it can infer the value of *C* given only the inputs *A* and *B*.

Four different training methods are compared: Using only synaptic plasticity, bookkeeping with and without pruning and finally training with the Gaussian convolution algorithm. Note that the Gaussian convolution algorithm uses pruning as well.

**Figure 8:**
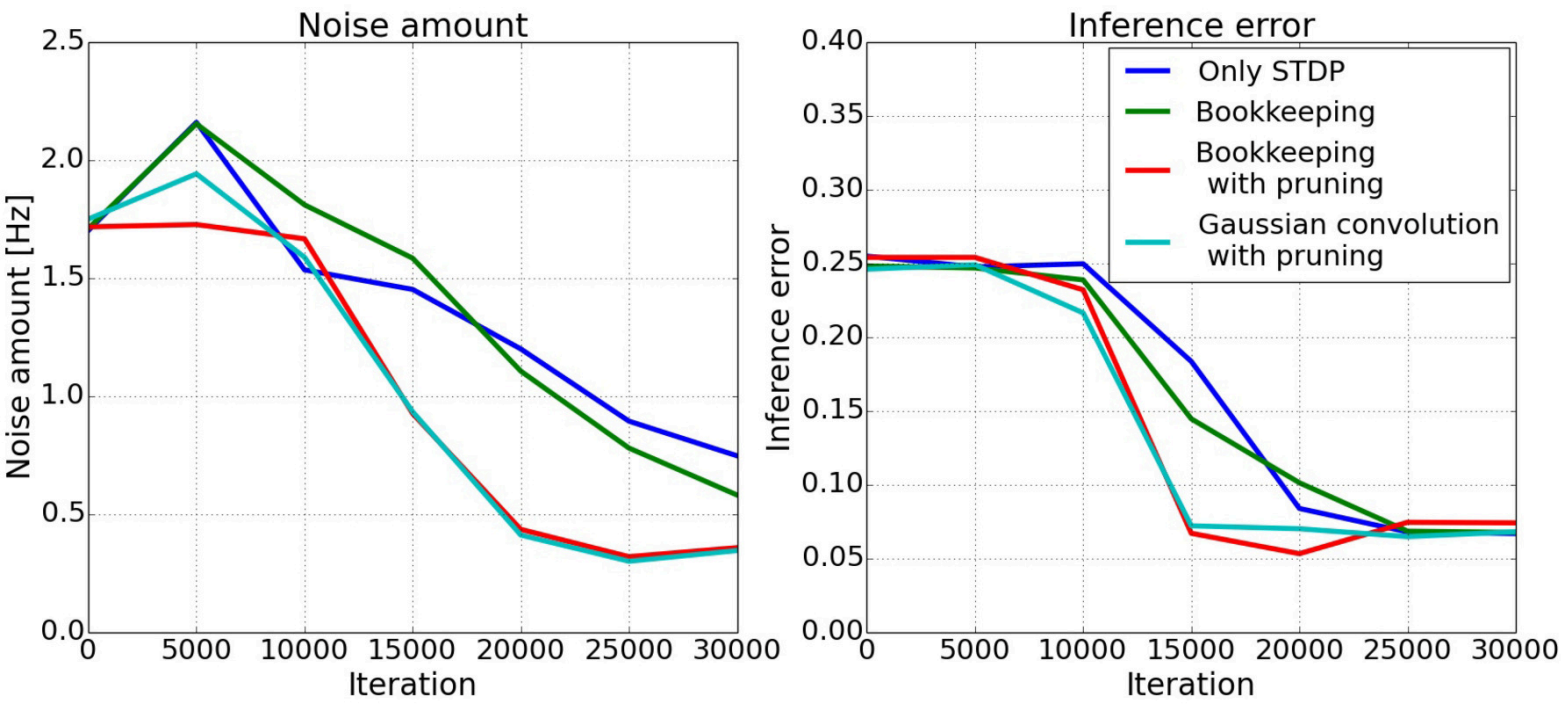
**Development of inferred responses in population ***C*****. The left plot shows the amount of noise *o*_noise_ in the spiking activity in population *C* of the three-way network. The two algorithms using pruning decrease the amount of noise faster. The right plot shows the error of the inference. The error is calculated as the difference of the mean of the spiking activity in *C* and the target value *A* + *B*. Clearly visible is the faster convergence of the bookkeeping with pruning and the Gaussian convolution algorithm.

The left plot in Figure 8 shows the amount of noise in the spiking activity in population *C* during testing where only *A* and *B* receive input. The actual error of the inference is shown in the right plot of Figure 8. The error is the difference of the target value *A* + *B* and the circular mean (Equation 11) of the spiking activity in population *C*. The two algorithms which decrease the total number of synapses converge faster to a lower error. It takes roughly 10,000–15,000 examples until the connections are trained enough for the spiking activity to change. The bookkeeping with decay and the Gaussian convolution algorithm learn faster, i.e., decrease their error faster, and achieve a lower amount of noise. These two algorithms have in common that they decrease the total number of synapses with pruning.

**Figure 9:**
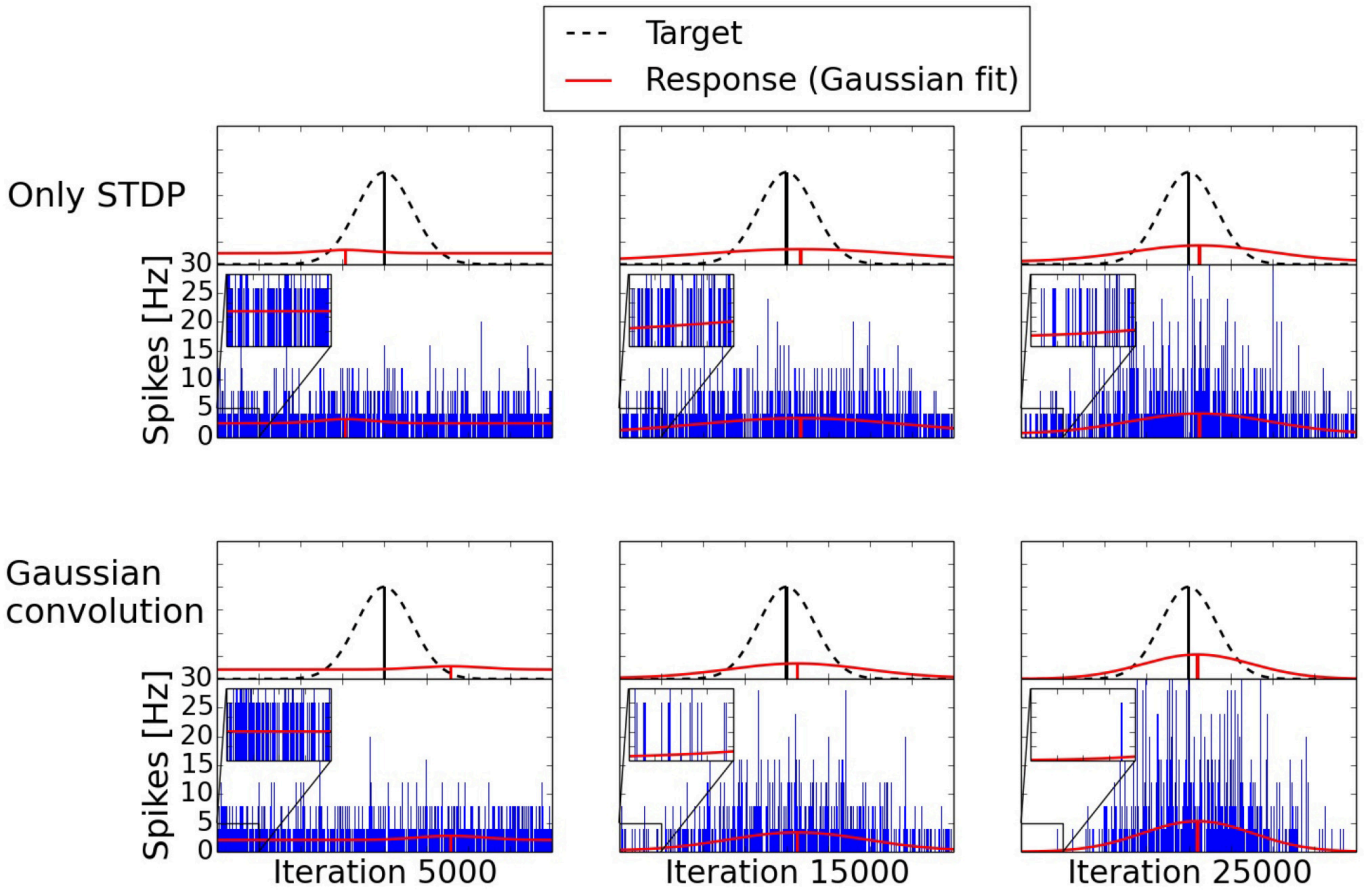
**Development of two three-way networks (with and without structural plasticity) over time**. Shown are their spike responses for the inference of a missing value. The upper row is a network trained only with STDP. The bottom row is trained with the Gaussian convolution algorithm and pruning. The blue bars are the spike responses of the individual neurons. The red Gaussians with offset are fitted to these responses. The dashed Gaussian represents the target value using the same standard deviation as the input of the two given inputs. With additional training the difference of the mean between the response and the target, the offset due to noise, and the standard deviation of the response decrease. But structural plasticity and pruning do so at a faster rate.

The development of the spiking activity for the inference can be seen in Figure 9. Two networks are compared after 5000, 15,000, and 25,000 shown input examples. The response after 5000 iterations is still mostly random for both networks. After 15,000 iterations the network trained with the Gaussian convolution algorithm and pruning produces a less noisy signal than the network trained only with STDP. With additional training both networks manage to produce a clearer Gaussian response. But structural plasticity and pruning improve the speed of the learning process by a large margin.

### 3.3. Structural plasticity preserves tuning

**Figure 10:**
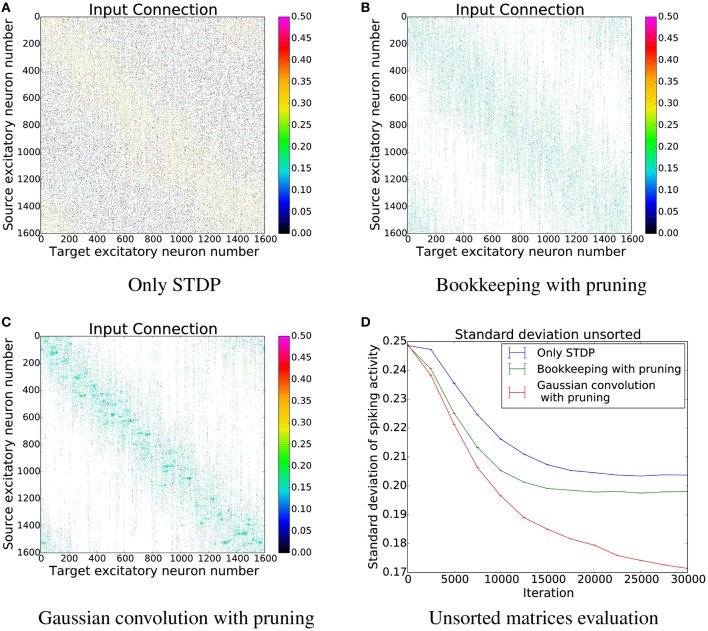
**The connection matrices from the input population to the computation population after training**. The columns of these matrices were only sorted once before the start of the simulation. **(D)** shows the performance of these unsorted matrices. The algorithm using Gaussian convolution achieves lower variance in the spike response thanks to the inclusion of spatial information. **(A)** Only STDP. **(B)** Bookkeeping with pruning. **(C)** Gaussian convolution with pruning. **(D)** Unsorted matrices evaluation.

In order to better understand the changes induced by using structural plasticity in addition to STDP, we also investigated how it affects the preferred input-tuning of neurons. Before starting learning, the columns of the initialization matrices were sorted such that neurons with strong weights to input neurons that are encoding low-values are at the beginning, and neurons with strong weights to high-values input neurons are at the end. We then simulated learning with different structural plasticity mechanisms (some of which use spatial information for the generation of new synapses) and without structural plasticity. The resulting matrices are shown in Figure 10.

The simulation that uses only STDP shows that the initial tuning of the neurons (which is due to fluctuations in the random initialization) is preserved to some extent and that the neurons preferred input-tuning after learning is influenced by its initial variations.

Including structural plasticity and pruning strongly increases the chances that initial preference of the input-tuning is preserved. This can be seen by observing that there are much less neurons that develop receptive fields that are not on the diagonal, i.e., that are different from their initial preference. The network trained with a spatial structural plasticity algorithm based on Gaussian convolution reinforces the initial tuning even stronger. Interestingly, the increased probability near already existing synapses also leads to the forming of patches of synapses.

## 4. Discussion

We simulate the process of structural plasticity using models with different underlying mechanisms and assumptions. The mechanisms ranged from simple deletion of the weakest synapses to more sophisticated monitoring of synapses and finally the inclusion of spatial information. Additionally, some implementations decrease the total number of synapses similarly to the pruning in the mammalian brain after peak synaptic density was achieved early in development (Huttenlocher, 1979; Navlakha et al., 2015). Two different network topologies were used to evaluate the performance of the algorithms. A smaller network to compare the noise amount of the responses with the different models and a bigger network that allowed us to compare the influence of the models on inference capabilities.

The results of the simulations show that structural plasticity can improve the learning process. Specifically, the noise in the response of the small network is reduced roughly 30% faster with structural plasticity. The inferred response in the big network is less noisy if a structural plasticity algorithm *with pruning* is used. The noise amount of the bookkeeping without pruning network is not significantly lower. This reduction of noise in the responses means that the networks are able to transmit the represented value with a clearer signal to connected populations.

This finding is especially interesting when connected to the results of the inference performance. Using structural plasticity with pruning reduces training time until the network reaches peak inference performance to about half of what is needed without pruning but without pruning the structural plasticity has little effect on learning speed. The positive results of synaptic pruning during training are in good agreement with (Navlakha et al., 2015).

Together those findings suggest that the fast reduction of the inference error and the decrease of noise in the response, which is facilitated by the structural plasticity (especially when combined with pruning), makes learning easier on a network level. Intuitively, if a population is confronted with less noisy inputs, fewer examples are needed to understand (or learn) the underlying relation between them.

As neuron populations in deep neural networks increase in size, established compression methods such as Huffman coding have lately found their way into the field of computational neuroscience for reducing the amount of data to be stored (Han et al., 2015). The use of structural plasticity as introduced here, can contribute to this effort by allowing control over the degree of sparseness in the network connectivity. Viewed under the light of lossy compression, the potential for not only data reduction but also data access reduction is given through the developed biologically inspired model.

To summarize, the addition of structural plasticity is an improvement to the current learning paradigm of only focusing on adjusting weight strengths rather than adjusting the actual connectivity.

### 4.1. Reducing simulation costs

While the results and the last subsection focused on improvements in terms of performance of the model, there is another very important aspect: Resource cost of the simulation. Practically the simulation of synapses requires a considerable amount of the total computation (Diehl and Cook, 2014) and poses a big challenge for implementation on neuromorphic hardware when the synapses are “hardwired” in silicon as in state-of-the-art analog VLSI spiking neuron processors (Qiao et al., 2015) The simulations presented here also benefited from reduced simulation times, i.e., a network trained with only STDP ran for 169 seconds^1^ to train on an additional 400 examples. Compared to a network which was trained with bookkeeping and pruning to reduce the number of synapses to roughly half of the starting amount which only ran for 125 seconds (roughly 25% faster). If the bookkeeping algorithm was used for the 400 examples an additional overhead of roughly 7 s brought the time to 132 s. Therefore keeping the number of synapses to a minimum is desirable. Of course this should ideally not impact the resulting performance negatively. But as shown here, there can even be a performance improvement by sensibly pruning synapses, mainly due to a reduction of the noise.

We can look at the “price-performance” of the model from different points of view. Firstly, we could fix a target accuracy and create a system that achieves the target accuracy while using as few synapses as possible. A practical scenario might be the need to implement natural language processing capabilities in a system with very stringent energy constraints like a mobile phone (Diehl et al., 2016a,b), where the systems needs a certain precision for it to be useful. After finding a small system with the required performance, it could be implemented in hardware and deployed on a device.

The second scenario is that the number of available synapses could be fixed while trying to optimize performance on that system, e.g., due to limited size of a neuromorphic device or limited time for simulation on traditional computer. If the low number of synapses is mainly needed for running a system after learning, it would be useful to start with a denser connectivity and apply pruning, and only implement the pruned network in the final system. However, as shown also using a constant number of synapses with structural plasticity potentially increases the raw performance while not leading to higher costs after training, which therefore also increases the price-performance of the model.

Therefore structural plasticity is also interesting for existing spiking networks that are designed to solve machine-learning tasks (Neftci et al., 2014; Zhao et al., 2014; Diehl and Cook, 2015) to not only increase their performance but also lower simulation cost.

### 4.2. Biological plausibility

Although the described work does not aim at reproducing biological effects in their highest level of detail, the underlying mechanisms of the introduced model take strong inspiration from the biological processes involved in the structural plasticity of the mammalian brain. These mechanisms were abstracted to an extent that it was possible to gain a computational advantage.

An example for this approach of trading off computational efficiency and biological plausibility is the exploitation of the finding that activity dependent reorganization of the biological brain shows observable effects over longer time windows than synaptic plasticity (hours to months) (Holtmaat and Svoboda, 2009). By discretization of the structural plasticity model in a second time-domain with time-steps that are large multiples of those of the synaptic plasticity time-domain, a trade-off between the introduced overhead of computations and the resulting optimization in the weight-matrix was made.

Similarly, the model purposely neglects the dynamics of receptor expression during synaptogenesis. Instead of introducing initially “silent” synapses that only hold NMDA receptors and can only be co-activated by neighbors on the same dendritic branch, here synapses allays perform STDP-like behavior. To obtain a comparable effect however, newly instantiated synapses were located close to those present synapses with the highest weight in the neuron which speeds up convergence to the learning goal.

As the main application of the introduced model is thought to be that of an optimization technique for established methods in spiking neural networks, here we begin training on a randomly initialized sparse connectivity-matrix instead of initializing with zero connectivity and including a process of activity independent synaptogenesis that simulates network development. This step not only greatly reduces computational overhead, it also allows to maintain a continuously fixed sparseness in the matrix which guarantees the reduction of memory utilization and can be seen as a form of lossy compression.

A further mechanism that the described model strongly relies on is synaptic scaling as a form of weight normalization. The implication here is that as popular synapses get potentiated by synaptic plasticity, the weight of less activated synapses tends to be depressed below the threshold which causes the vulnerability to pruning. A biological foundation behind this mechanism is a NMDA-mediated heterosynaptic depression that accompanies longterm potentiation (Royer and Paré, 2003).

## Author contributions

All authors contributed to the concept. RS and PD did the simulations. RS, PD, and RG wrote the paper.

## Funding

PD was supported by the SNF Grant 200021-143337 “Adaptive Relational Networks.” RG has received funding from the European Union Seventh Framework Programme (FP7/2007- 2013) under grant agreement no. 612058 (RAMP).

### Conflict of interest statement

The authors declare that the research was conducted in the absence of any commercial or financial relationships that could be construed as a potential conflict of interest.

## Footnotes

^1^These speed evaluations were done single threaded on a i7-3520M 2.90 Ghz and 8 GB RAM
